# Determinants of mortality in hypertensive patients admitted with COVID-19: a single-centre retrospective study at a tertiary hospital in South Africa

**DOI:** 10.1186/s12872-024-03964-2

**Published:** 2024-06-10

**Authors:** Ahmed Sameer Ikram, Somasundram Pillay

**Affiliations:** 1 University of KwaZulu-Natal, Durban, South Africa; 2 Internal Medicine, Division of Internal Medicine, School of Clinical Medicine, College of Health Sciences, University of KwaZulu-Natal, Durban, South Africa

**Keywords:** COVID-19, Hypertension, Mortality, South Africa, Hypertension-mediated organ damage (HMOD), Risk factors

## Abstract

**Background:**

The Coronavirus Disease 2019 (COVID-19) pandemic has significantly impacted global health, with successive outbreaks leading to substantial morbidity and mortality. Hypertension, a leading cause of cardiovascular disease globally, has been identified as a critical comorbidity in patients with severe COVID-19, exacerbating the risk of adverse outcomes. This study aimed to elucidate the impact of hypertension on COVID-19 outcomes within the South African context.

**Methods:**

A retrospective analysis was conducted at King Edward VIII Hospital, KwaZulu-Natal, South Africa, encompassing patients aged 13 years and above admitted with laboratory-confirmed SARS-CoV-2 infection between June 2020 and December 2021. The study investigated the association between hypertension and COVID-19 outcomes, analysing demographic, clinical, and laboratory data. Statistical analysis involved univariate and multivariate logistic regression to identify predictors of mortality among the hypertensive cohort.

**Results:**

The study included 420 participants—encompassing 205 with hypertension. Hypertensive patients demonstrated significantly greater requirements for oxygen and steroid therapy (*p* < 0.001), as well as higher mortality rates (44.88%, *p* < 0.001)) compared to their non-hypertensive counterparts. Key findings demonstrated that a lower oxygen saturation (adjusted odds ratio (aOR) 0.934, *p* = 0.006), higher pulse pressure (aOR 1.046, *p* = 0.021), elevated CRP (aOR 1.007, *p* = 0.004) and the necessity for mechanical ventilation (aOR 5.165, *p* = 0.004) were independent risk factors for mortality in hypertensive COVID-19 patients. Notably, the study highlighted the pronounced impact of hypertension-mediated organ damage (HMOD) on patient outcomes, with ischemic heart disease being significantly associated with increased mortality (aOR 8.712, *p* = 0.033).

**Conclusion:**

Hypertension significantly exacerbates the severity and mortality risk of COVID-19 in the South African setting, underscoring the need for early identification and targeted management of hypertensive patients. This study contributes to the understanding of the interplay between hypertension and COVID-19 outcomes, emphasising the importance of considering comorbidities in the management and treatment strategies for COVID-19. Enhanced pandemic preparedness and healthcare resource allocation are crucial to mitigate the compounded risk presented by these concurrent health crises.

## Background

The global health landscape continues to be significantly impacted by Coronavirus Disease 2019 (COVID-19), with many countries, including South Africa, having faced successive outbreaks that have claimed millions of lives worldwide [1]. Despite a noticeable decline in both the incidence and fatality rates, the advent of new virus strains—characterized by their varied behaviour and uncertain response to vaccines—underscores the importance of maintaining alertness [2]. Consequently, there has been a concerted effort to identify predictors of adverse outcomes, among which hypertension has been recognised as a critical factor.

Hypertension is the leading cause of cardiovascular disease and premature death globally. Despite widespread disease awareness and treatment availability, the prevalence of hypertension has risen – predominantly in low- and middle-income countries (LMICs); representing a vulnerable population [3].

With global research, the association between hypertension and COVID-19 has become clearer. Not only is hypertension a significant comorbidity in patients with severe COVID-19, most studies demonstrate an elevated risk of severe disease and mortality in hypertensive patients hospitalised with COVID-19 [4–10]. Blood pressure control during the course of the COVID-19 illness also appears to play a role in predicting morbidity [11]. Ran et al. investigated 803 hypertensive patients with COVID-19 and demonstrated a significant association between average and increased systolic blood pressure variability with adverse outcomes in these patients [11]. An elevated average systolic blood pressure was found to be a significant predictor of heart failure development while high blood pressure variability was found to be associated with increased mortality and ICU admission – underpinning the importance of maintenance of a stable blood pressure [11]. One theory proposes that upregulation of the renin-angiotensin system and increased expression of angiotensin converting enzyme-2 (ACE-2) in hypertensive patients with COVID-19 may potentially facilitate virus entry into target cells and result in more severe disease [12].

In the African context, the exploration of hypertension's impact on COVID-19 outcomes remains limited. A comprehensive systematic review and meta-analysis focusing on sub-Saharan Africa uncovered a notable increase in mortality risk (OR 1.32; 95% CI 1.13 – 1.50) for hypertensive patients afflicted with COVID-19 [12]. This analysis spanned across eight sub-Saharan nations, incorporating data from 29,945 patients, with a significant contribution from a South African cohort of 22,308 patients [12, 13]. A separate extensive study in South Africa, involving 219,265 COVID-19-hospitalized individuals, identified hypertension as the most common underlying condition—present in 38.6% of cases [14]. This study also highlighted a significant association between black race and hypertension with increased mortality rates within the hospital setting. Interestingly, a distinct variation was noted in the association of hypertension with mortality between different health sectors—it was significantly linked to mortality in the private sector but not in the public sector. Furthermore, there was a stronger effect of race and comorbidities observed in the private sector—suggesting nuanced differences in the impact of these demographical factors across these sectors [14].

Amid extensive research into the link between hypertension and COVID-19, there remains a notable scarcity of data specifically pertaining to African populations. This gap is significant considering the distinct racial disparities evident in the prevalence and severity of hypertension [15, 16]. Among different racial groups, black individuals exhibit the highest rates of hypertension, which tends to be more severe and less responsive to conventional treatments [15]. This demographic is also disproportionately affected by an increased risk of stroke, heart failure, and kidney disease [16]. Furthermore, several studies demonstrate greater rates of SARS-CoV-2 infection and disease severity in black patients compared to their white counterparts. A cohort of 3626 patients in Louisiana revealed that black patients comprised 76.9% of the patients that were hospitalised and 70.6% of the patients that demised, despite only making up 31% of the local population [17]. This was attributed to a higher prevalence of chronic conditions and socioeconomic factors, including differences in the types of jobs that may increase community exposure [17]. This racial disparity was corroborated by a large systematic review and meta-analysis comprising more than 4 million patients; again demonstrating higher rates of infection and severe disease in ethnic minorities [18]. Socioeconomic determinants were again found to be strongly associated with COVID-19 outcomes in these groups [18].

Given the unique genetic makeup of African individuals, together with resource and healthcare limitations, research conducted globally is not necessarily generalisable to the African context, and underscores the necessity for research that is tailored to the local milieu. This urgency is amplified by the growing incidence of hypertension across sub-Saharan Africa and other low- and middle-income regions, coupled with the dire implications of potential new surges of SARS-CoV-2 infections. This study sought to fill the existing gap in knowledge by exploring the link between hypertension and COVID-19 within the South African setting and, secondarily, to compare our insights with global findings. To the best of our knowledge, this will be the first study specifically investigating the association between hypertension and COVID-19 in the South African context. In addition to contributing to the local knowledge pool, the data gained will also be able to guide local policies and guidelines, thereby enhancing preparedness and response strategies.

## Methods

### Aim

This study aimed to evaluate the association between hypertension and COVID-19 within a predominantly African population, and secondarily to compare our findings to those observed globally.

### Study design and setting:

A retrospective cohort study was conducted at King Edward VIII Hospital (KEH)—a tertiary institution situated in KwaZulu-Natal, South Africa.

### Participant selection:

The study encompassed all individuals aged 13 years and above who were admitted to KEH with a laboratory-confirmed diagnosis of SARS-CoV-2 infection between 1 June 2020 and 31 December 2021. The primary exclusions included patient medical records lacking necessary information and pregnant individuals.

A sample size of 200 patients with COVID-19 was required to estimate the proportion of patients with hypertension to within ± 10% with a probability of 95% and a non-informative baseline of 50%. If 40% of patients have hypertension, this would result in: 80 with hypertension and 120 without hypertension. A 20% difference in mortality would then be required for statistical significance with a probability of 95% and power of 80% and assuming a 25% mortality in the normal group. To improve generalisability, the sample size was increased to 420 participants. Stata v17 was used for the sample size calculation.

### Data collection and ethical approval

The University of KwaZulu-Natal Biomedical Research and Ethics Committee (BREC) (BREC/00005457/2023) granted ethical clearance for this study, with additional approval from the Department of Health and the respective site (KEH). To ensure privacy, data was anonymised before analysis. Given the study's retrospective nature, the requirement for informed consent was waived by the Biomedical Research and Ethics Committee. All research activities adhered strictly to applicable guidelines and regulatory standards.

The following data was obtained from medical records of participants, and analysed as per aims of the study:

1. Age (years)
2. Gender
  ▪ Male
  ▪ Female
3. Race
  ▪ Black African
  ▪ White
  ▪ Mixed
  ▪ Asian
4. Presence of comorbidities:
  ▪ Diabetes mellitus
  ▪ Hypertension
  ▪ HIV
  ▪ Asthma/COPD
  ▪ Chronic Kidney Disease
5. Presence and type of hypertension-mediated organ damage (HMOD)
6. Admission clinical parameters:
  ▪ Oxygen saturation (%)
  ▪ Respiratory rate (breaths/minute)
  ▪ Heart rate (beats/minute)
  ▪ Systolic blood pressure (mmHg)
  ▪ Diastolic blood pressure (mmHg)
  ▪ Pulse pressure (mmHg)
  ▪ Random glucose (mmol/l)
  ▪ Temperature (˚C)
7. Laboratory variables:
  ▪ White cell count (× 10^9^/l)
  ▪ C-reactive protein (mg/l)
8. Treatment:
  ▪ Required steroid therapy
  ▪ Required supplemental oxygen
  ▪ Required mechanical ventilation
9. Outcome:
  ▪ Survivor
  ▪ Non-survivor

### Statistical analysis

The data collected was analysed with SPSS version 27.0 and Stata v17. The Shapiro–Wilk test was used to determine the distribution of data. Quantitative data was presented as mean (standard deviation) (SD) or median (interquartile range)(IQR) depending on the distribution of the data, and compared using Student’s t-test or Mann–Whitney U-test respectively. Categorical data was presented as frequencies and percentages and compared using Chi squared tests or Fisher’s exact test where frequencies were small. Univariate and multivariate logistic regression was undertaken to identify predictors of mortality in the hypertensive cohort. A log rank test was then performed to evaluate the difference in survival rates for those with and without hypertension. A *p* value of < 0.05 was regarded as statistically significant.

### Study procedure

The sample population was divided into those with and without hypertension with comparisons made between the two groups in terms of demographics, clinical and laboratory features, requirement for steroid therapy, oxygen therapy and/or mechanical ventilation, and outcome. The hypertension cohort was further divided into survivors and non-survivors and comparisons made between the two cohorts to identify risk factors for mortality. Blood pressure was measured using standardised automatic blood pressure devices with size-appropriate arm cuffs. The primary outcome of intertest was in-patient mortality.

### Definitions

COVID-19 was defined by a positive SARS-CoV-2 polymerase chain reaction (PCR) laboratory result. A non-survivor was defined as a participant that demised during period of hospital stay, whereas a survivor was defined as a participant that was either discharged home, down referred, or up referred without demise during period of hospital admission. Patients with hypertension were defined as those participants already on anti-hypertensive therapy on admission, or those with an elevated blood pressure ≥ 140/90 mmHg during hospital admission and requiring initiation of anti-hypertensive therapy. The presence and type of hypertension-mediated organ damage (HMOD) was based on previous documentation as well as current presentation as follows:

- Cerebrovascular disease – CT imaging supporting the diagnosis.
- Retinopathy – documentation of fundoscopic findings in keeping with hypertensive retinopathy.
- Ischemic heart disease – self-reported history of treatment for angina or previous acute coronary syndrome; diagnostic testing (exercise ECG, myocardial perfusion scan or coronary angiogram); or previous revascularisation procedure (thrombolysis or percutaneous coronary intervention).
- Chronic kidney disease – documentation of an estimated GFR < 60 ml/min/1.73m^2^, measured using the CKD-EPI equation, for ≥ 3 months.
- Peripheral vascular disease – self-reported history of intermittent claudication and/or ischemic rest pain; clinical examination findings that reveal diminished lower extremity pulses, ischemic ulcers, gangrene or an ankle brachial index < 0.9; diagnostic imaging (CT angiogram); or previous invasive intervention for peripheral vascular disease (revascularisation procedure or amputation).

## Results

A total of 420 participants with laboratory-confirmed SARS-CoV-2 infection were enrolled in the study – comprising 205 with hypertension and 215 without hypertension. The median age of those with hypertension was 61 (52–69) compared to 45 (32–57) for those without hypertension (*p* < 0.001). The demographic profile of the sample comprised predominantly Black Africans with a well-balanced gender representation. Gender and race distribution did not exhibit any statistical significance; however, hypertension demonstrated a strong association with several comorbidities. Notably, diabetes, HIV and chronic kidney disease were significantly more prevalent in participants with hypertension. Moreover, participants with hypertension were significantly more likely to receive steroid therapy (*p* < 0.001), oxygen therapy (*p* < 0.001), and demise (*p* < 0.001) compared to their non-hypertensive counterparts (Table 1).

**Table 1:** Demographics, comorbidities, treatment and outcome – comparison between those with and without hypertension

		Total (*n* = 420)	With Hypertension (*n* = 205)	Without Hypertension (*n* = 215)	*p* value
		f (%)	f (%)	f (%)	
Age (years)	10–19	7 (1.67)	1 (0.49)	6 (2.79)	< 0.001
	20–29	40 (9.52)	4 (1.95)	36 (16.74)	
	30–39	53 (12.62)	9 (4.39)	44 (20.47)	
	40–49	68 (16.19)	20 (9.76)	48 (22.33)	
	50–59	100 (23.81)	60 (29.27)	40 (18.61)	
	60–69	88 (20.95)	62 (30.24)	26 (12.09)	
	70–79	48 (11.43)	37 (18.05)	11 (5.12)	
	80–89	13 (3.10)	9 (4.39)	4 (1.86)	
	90–99	3 (0.71)	3 (1.46)	0 (0)	
	Total	420	205	215	
Gender	Female	230 (54.76)	120 (58.54)	110 (51.16)	0.129
	Male	190 (45.24)	85 (41.46)	105 (48.84)	
	Total	420	205	215	
Race	Black African	354 (84.05)	165 (80.49)	188 (87.44)	0.198
	White	26 (6.19)	16 (7.80)	10 (4.65)	
	Mixed	3 (0.71)	1 (0.49)	2 (0.93)	
	Asian	38 (9.05)	23 (11.22)	15 (6.98)	
	Total	420	205	215	
Comorbidities	Diabetes	149 (35.48)	112 (54.63)	37 (17.21)	< 0.001
	COPD/Asthma	29 (6.90)	13 (6.34)	16 (7.44)	0.657
	HIV	122 (29.05)	46 (22.44)	76 (35.35)	0.004
	Chronic Kidney Disease	47 (11.19)	35 (17.07)	12 (5.58)	0.001
Required Steroid Therapy	Yes	284 (67.62)	167 (81.46)	117 (54.42)	< 0.001
	No	136 (32.38)	38 (18.54)	98 (45.58)	
	Total	420	205	215	
Required Oxygen Therapy	Yes	289 (68.81)	169 (82.44)	120 (55.81)	< 0.001
	No	131 (31.19)	36 (17.56)	95 (44.19)	
	Total	420	205	215	
Required Mechanical Ventilation	Yes	83 (19.76)	45 (21.95)	38 (17.67)	0.271
	No	337 (80.24)	160 (78.05)	177 (82.33)	
	Total	420	205	215	
Outcome	Non-survivor	129 (30.71)	92 (44.88)	37 (17.21)	< 0.001
	Survivor	291 (69.29)	113 (55.12)	178 (82.79)	
	Total	420	205	215	

In our study, analysis of clinical parameters on admission demonstrated significant disparities in individuals with and without hypertension. Of note, the former group had a significantly higher respiratory rate, heart rate, systolic blood pressure, diastolic blood pressure, pulse pressure and glucose; as well as a lower oxygen saturation (Table 2).

**Table 2:** Age, admission vital signs and laboratory variables – comparison between those with and without hypertension

	Total (*n* = 420)		With Hypertension (*n* = 205)		Without Hypertension (*n* = 215)		*p* value
	Median (IQR)	Mean (SD)	Median (IQR)	Mean (SD)	Median (IQR)	Mean (SD)	
Age (years)	53.5 (41–64.5)		61 (52–69)		45 (32–57)		< 0.001
Oxygen Saturation (%)	93 (86–98)		89 (80–96)		96 (90–99)		< 0.001
Respiratory rate (breaths/minute)	21 (20–24)		22 (20–25)		20 (20–23)		< 0.001
Heart rate (bpm)	94 (84–106)		97 (87–110)		92 (84–101)		0.005
Systolic blood pressure (mmHg)	125 (113–139)		130 (118–145)		121 (110–132)		< 0.001
Diastolic blood pressure (mmHg)		76.03 (13.11)		78.12 (14.67)		74.04 (11.1)	0.001
Pulse pressure (mmHg)	48.5 (40–60)		52 (43–64)		47 (40–55)		< 0.001
Temperature (ºC)	36.6 (36.4–36.7)		36.5 (36.4–36.7)		36.6 (36.4–36.8)		0.022
Glucose (mmol/l)	7.8 (5.85–12.1)		9.6 (7.1–15)		6.7 (5.5–8.9)		< 0.001
White cell count (WCC) (× 10^9^/l)	8.3 (6.1–11.1)		9.6 (6.9–12.3)		7.4 (5.6–9.7)		< 0.001
C-reactive protein (CRP) (mg/l)	100 (25.5–167)		120 (59–191)		63 (12–143)		< 0.001

Similarly, significant differences were noted in admission laboratory markers – with a higher white cell count [9.6 × 10^9^/l (6.9 – 12.3) vs. 7.4 × 10^9^/l (5.6 – 9.7); *p* < 0.001] and CRP [120 mg/l (59–191) vs 63 mg/l (12–143); *p* < 0.001) in those with hypertension.

The total cohort compromised 205 participants with hypertension. We aimed to delineate factors associated with death in those with hypertension—thus, this group was subdivided based on outcome into survivors and non-survivors (Table 3).

**Table 3:** Patients with hypertension – comparison between survivors and non-survivors (categorical variables)

		Total (*n* = 205)	Survivors (*n* = 113)	Non-survivors (*n* = 92)	*p* value
		f (%)	f (%)	f (%)	
Age (years)	10–19	1 (0.49)	1 (0.88)	0 (0)	0.301
	20–29	4 (1.95)	2 (1.77)	2 (2.17)	
	30–39	9 (4.39)	7 (6.19)	2 (2.17)	
	40–49	20 (9.76)	12 (10.62)	8 (8.7)	
	50–59	60 (29.27)	37 (32.74)	23 (25)	
	60–69	62 (30.24)	28 (24.78)	34 (36.96)	
	70–79	37 (18.05)	18 (15.93)	19 (20.65)	
	80–89	9 (4.39)	7 (6.19)	2 (2.17)	
	90–99	3 (1.46)	1 (0.88)	2 (2.17)	
	Total	205	113	92	
Gender	Female	120 (58.54)	69 (61.06)	51 (55.43)	0.416
	Male	85 (41.46)	44 (38.94)	41 (44.57)	
	Total	205	113	92	
Race	Black African	165 (80.49)	96 (84.96)	69 (75)	0.199
	White	16 (7.8)	7 (6.19)	9 (9.78)	
	Mixed	3 (1.46)	1 (0.88)	0 (0)	
	Asian	23 (11.22)	9 (7.96)	14 (15.22)	
	Total	205	113	92	
Comorbidities	Diabetes	112 (54.63)	57 (50.44)	55 (59.78)	0.182
	COPD/Asthma	13 (6.34)	7 (6.19)	6 (6.52)	0.924
	HIV	46 (22.44)	29 (25.66)	17 (18.48)	0.220
Required Steroid Therapy	Yes	167 (81.46)	78 (69.03)	89 (96.74)	< 0.001
	No	38 (18.54)	35 (30.97)	3 (3.26)	
	Total	205	113	92	
Required Oxygen Therapy	Yes	169 (82.44)	80 (70.8)	89 (96.74)	< 0.001
	No	36 (17.56)	33 (29.2)	3 (3.26)	
	Total	205	113	92	
Required Mechanical Ventilation	Yes	45 (21.95)	7 (6.19)	38 (41.3)	< 0.001
	No	160 (78.05)	106 (93.81)	54 (58.7)	
	Total	205	113	92	
HMOD	Yes	84 (40.98)	27 (23.89)	57 (61.96)	< 0.001
	No	121 (59.02)	86 (76.11)	35 (38.04)	
	Total	205	113	92	
Number of target organs affected	0	121 (59.02)	86 (76.11)	35 (38.04)	< 0.001
	1	71 (34.63)	25 (22.12)	46 (50)	
	2	10 (4.88)	1 (0.88)	9 (9.78)	
	3	3 (1.46)	1 (0.88)	2 (2.17)	
	Total	205	113	92	
Specific target organ affected	Cerebrovascular disease	14 (6.83)	4 (3.54)	10 (10.87)	0.039
	Retinopathy	5 (2.44)	1 (0.88)	4 (4.35)	0.176
	Ischemic heart disease	33 (16.1)	7 (6.19)	26 (28.26)	< 0.001
	Nephropathy	35 (17.07)	11 (9.73)	24 (26.09)	0.002
	Peripheral vascular disease	13 (6.34)	7 (6.19)	6 (6.52)	0.924

The hypertensive cohort had an overall mortality of 44.88% (*n* = 92) with the non-survivor group having a significantly higher prevalence of receiving steroid therapy (*p* < 0.001), oxygen therapy (*p* < 0.001), and mechanical ventilation (*p* < 0.001).

In addition, we analysed the influence of hypertension-mediated organ damage (HMOD) on survival. Significant differences were observed between the cohorts with the non-survivor group having more participants with HMOD (*p* < 0.001). Furthermore, the number of target organs affected demonstrated significant associations with survival outcomes; survivors predominantly exhibited fewer affected target sites, whereas non-survivors had a greater number of target organs affected (*p* < 0.001). The specific target site affected also demonstrated notable differences – cerebrovascular disease (*p* = 0.039), ischemic heart disease (< 0.001) and nephropathy (0.002) were significantly more prevalent in the non-survivor group.

Further analysis of admission clinical and laboratory features amongst the hypertensive cohort revealed significant differences between survivors and non-survivors. The non-survivor group had a significantly higher median respiratory rate, heart rate, systolic blood pressure, pulse pressure, glucose, white cell count and CRP; and a significantly lower median oxygen saturation compared to non-survivors (Table 4).

**Table 4:** Patients with hypertension – comparison between survivors and non-survivors (continuous variables)

	Total (*n* = 205)		Survivors (*n* = 113)		Non-survivors (*n* = 92)		*p* value
	Median (IQR)	Mean (SD)	Median (IQR)	Mean (SD)	Median (IQR)	Mean (SD)	
Age (years)	61 (52–69)		59 (50–67)		63 (54–69.5)		0.112
Oxygen Saturation (%)	89 (80–96)		94 (88–98)		82 (73–88)		< 0.001
Respiratory rate (breaths/minute)	22 (20–25)		20 (20–23)		24 (21.5–26)		< 0.001
Heart rate (bpm)	97 (87–110)		90 (84–105)		104.5 (89.5–115.5)		< 0.001
Systolic blood pressure (mmHg)	130 (118–145)		128 (118–140)		138 (119–154.5)		0.031
Diastolic blood pressure (mmHg)		78.12 (14.67)		78.96 (12.54)		77.1 (16.95)	0.369
Pulse pressure (mmHg)	52 (43–64)		49 (40–58)		56.5 (44–69)		< 0.001
Temperature (ºC)	36.5 (36.4–36.7)		36.5 (36.4–36.7)		36.5 (36.4–36.7)		0.968
Glucose (mmol/l)	9.6 (7.1–15)		7.9 (6.2–12.6)		11.9 (8.8–17.55)		< 0.001
White cell count (WCC) (× 10^9^/l)	9.6 (6.9–12.3)		8.3 (6.5–10.8)		11.1 (8–15.4)		< 0.001
C-reactive protein (CRP) (mg/l)	120 (59–191)		92 (30–154)		155 (109.5–248.5)		< 0.001

Univariate logistic regression was undertaken on all predictor variables to identify significant associations with death in the hypertensive cohort. The significant variables then underwent multivariate logistic regression analysis (Table 5).

**Table 5:** Predictors of mortality in the hypertensive cohort—univariate and multivariate logistic regression analysis

Variable	Univariate analysis			Multivariate analysis		
	Unadjusted OR	95% CI	*p* value	Adjusted OR	95% CI	*p* value
Glucose	1.084	1.035 – 1.136	0.001	1.013	0.952 – 1.079	0.683
SBP	1.013	1.0003 – 1.027	0.045	1.013	0.960 – 1.014	0.342
Pulse pressure	1.034	1.014 – 1.054	0.001	1.046	1.007 – 1.087	0.021
Heart rate	1.035	1.017 – 1.054	< 0.001	1.014	0.988 – 1.039	0.298
Respiratory rate	1.133	1.062 – 1.209	< 0.001	1.004	0.925 – 1.089	0.928
Oxygen saturation	0.890	0.858 – 0.924	< 0.001	0.934	0.889 – 0.980	0.006
White cell count	1.103	1.040 – 1.171	0.001	0.970	0.898 – 1.047	0.436
C-reactive protein	1.009	1.005 – 1.012	< 0.001	1.007	1.002 – 1.011	0.004
Required steroid therapy	13.312	3.939 – 44.984	< 0.001	0.985	0.144 – 6.752	0.988
Required oxygen therapy	12.238	3.613 – 41.445	< 0.001	5.795	0.657 – 51.123	0.144
Required mechanical ventilation	10.656	4.463 – 25.440	< 0.001	5.165	1.682 – 15.858	0.004
Cerebrovascular disease	3.323	1.007 – 10.972	0.049	10.688	0.977 – 116.972	0.052
Ischemic heart disease	5.965	2.451 – 14.571	< 0.001	8.712	1.190 – 63.760	0.033
Nephropathy	3.273	1.505 – 7.117	0.003	6.894	0.947 – 50.180	0.057
Presence of HMOD	5.187	2.837 – 9.484	< 0.001	0.804	0.123 – 5.279	0.820

Among the clinical variables, pulse pressure and oxygen saturation were found to be significant after multivariate analysis, with a lower oxygen saturation (aOR 0.934; *p* = 0.006) and higher pulse pressure (aOR 1.046; *p* = 0.021) favouring mortality. In addition, hypertensive participants with ischemic heart disease were significantly more likely to demise (aOR 8.712; *p* = 0.033), as were those that received mechanical ventilation (aOR 5.165; *p* = 0.004).

From the laboratory markers, white cell count lost its statistical significance after multivariate analysis, whereas an elevated CRP still favoured mortality (aOR 1.007; *p* = 0.004).

**Fig. 1:**
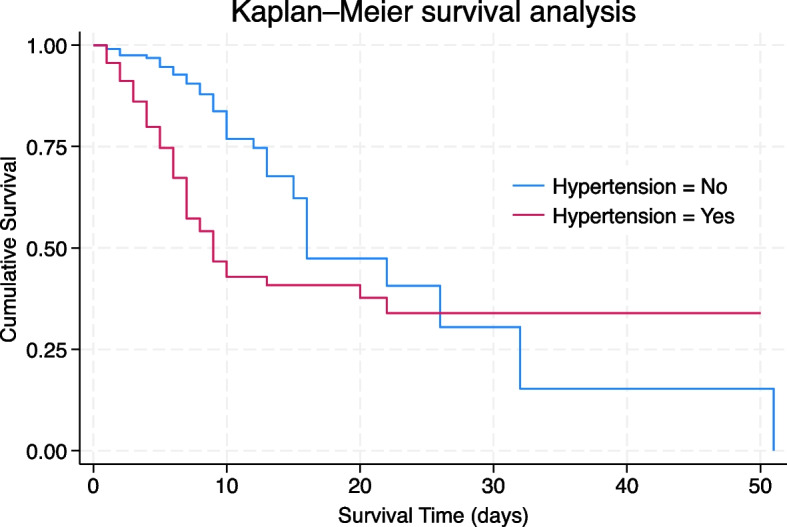
Kaplan Meier Survival Analysis – time to death (days) for participants with vs. without hypertension

Figure 1 demonstrates the difference in survival rates amongst those with and without hypertension. A log rank test revealed significantly lower survival rates in those with hypertension (*p* < 0.001), with a median time to death of 9 days. In contrast, those without hypertension had a median time to death of 16 days.

## Discussion

This study highlights the impact of hypertension on COVID-19 outcomes within the South African context—underscoring hypertension as a significant comorbidity that exacerbates the severity of COVID-19. In our study, the hypertensive cohort had significantly more individuals with clinical and laboratory markers of severity, a greater requirement for oxygen and steroid therapy, as well as a higher mortality rate compared to their non-hypertensive counterparts. Additionally, a lower oxygen saturation, higher pulse pressure, higher CRP, a requirement for mechanical ventilation, as well as the presence of ischemic heart disease were all found to be independent risk factors for in-hospital mortality amongst the hypertensive patients admitted with SARS-CoV-2 infection.

Despite an overall decline in SARS-CoV-2 infections worldwide – it remains a matter of great interest given the lethality of the previous pandemic, together with the potential for resurgence of mutated strains of this virus with variant behaviour and unpredictable vaccine response. Parallelly, hypertension emerges as a leading contributor to cardiovascular diseases and premature mortality worldwide, with its prevalence notably increasing in low- and middle-income countries (LMICs) [3]. This confluence poses the risk of intersecting pandemics. While international research has underscored the relationship between hypertension and the severity and outcomes of COVID-19, these findings are not necessarily generalisable to African populations given the unique phenotype of hypertension in black patients combined with healthcare and resource limitations [15, 16]. As such, our study aimed to better delineate the association between hypertension and COVID-19 within the local context in a predominantly black population, and secondarily to compare our findings with those found globally. Our hope is that the data obtained will contribute positively to pandemic preparedness and assist in mitigating fatalities associated with these two conditions.

Despite robust global research, there is a paucity of data from the African continent. To the best of our knowledge, this is the first African study investigating risk factors for mortality in hypertensive patients hospitalized with COVID-19.

Our study demonstrated an increased likelihood of severe disease and mortality in hypertensive patients admitted with COVID-19 as compared to non-hypertensives. This finding aligns with a comprehensive systematic review and meta-analysis involving 29,945 patients across eight sub-Saharan African countries, and is supported by a large-scale South African study by Jassat et al., which identified a significant link between hypertension and mortality among 219,265 COVID-19 hospitalized patients [12, 14]. Notably, Jassat et al. found this association primarily in the private healthcare sector, unlike our study, which observed a significant relationship despite being conducted at a public hospital setting [14]. Various theories have been proposed to explain the heightened risk for severe disease and mortality in hypertensive COVID-19 patients. One such theory focuses on the role of angiotensin converting enzyme-2 (ACE-2). It is hypothesized that the upregulation of the renin-angiotensin system in individuals with arterial hypertension leads to increased ACE-2 expression on various organ surfaces such as the heart, lungs and kidneys [12]. This effect might be amplified by the use of angiotensin receptor blockers (ARBs) or angiotensin converting enzyme inhibitors (ACEIs), further increasing ACE-2 expression and facilitating virus entry into target cells—potentially resulting in more severe disease [12]. Additionally, the black population with hypertension often presents with more severe and resistant forms of the condition and a higher prevalence of target organ damage, categorizing them as particularly vulnerable to severe COVID-19 outcomes [15].

A lower oxygen saturation on admission was found to be an independent risk factor for mortality amongst patients with hypertension—for every one percent increase in oxygen saturation, the odds of mortality decreased by 6.6%. This readily accessible clinical marker, available at the initial evaluation, could significantly aid in the early stratification of risk among this patient group. Our results resonate with a Chinese study by Yang et al., which also identified reduced oxygen saturation as a standalone predictor of increased mortality risk in hypertensive patients hospitalized due to COVID-19 [19].

A higher admission pulse pressure was found to be an independent risk factor for mortality in hypertensive patients in our study. This may serve as a marker of arterial stiffness – a well-documented outcome of longstanding arterial hypertension and risk factor for death even in patients without COVID-19 [20]. An elevated systolic blood pressure increases myocardial oxygen demand and predisposes to vascular injury, whereas a lower diastolic blood pressure may be a limiting factor for coronary perfusion [20]. Using pulse pressure as a proxy for arterial stiffness, the hypertensives in our study with increasing levels of arterial stiffness were more likely to demise – with a 46% greater likelihood of death for every 10 mmHg increase in pulse pressure. This is in keeping with a large multi-center Spanish study by Rodilla et al. comprising 12,170 participants whereby arterial stiffness, defined as a pulse pressure ≥ 60 mmHg, had independent prognostic value for all-cause mortality in hospitalized patients with COVID-19 [21]. Advanced age is another major determinant of arterial stiffness; however, pulse pressure retained its statistical significance in our study even after adjusting for age and other confounders. Interestingly, the pulse pressure difference noted between survivors and non-survivors in the hypertensive cohort was mainly due to higher systolic blood pressures in the latter group; whereas there was no statistically significant difference in diastolic blood pressure between survivors and non-survivors.

The prognostic value of C-reactive protein (CRP) in hypertensive patients with COVID-19 remains underexplored both globally and in the local context. In our study, an elevated CRP on admission was identified to be an independent risk factor for mortality—possibly reflecting the extent of the cytokine storm and serving as a laboratory marker of disease severity. For every 10 mg/l increase in CRP, the likelihood of mortality increased by 7% in those with hypertension. Studies globally demonstrate an elevated risk of severe disease and adverse outcome in individuals with COVID-19 with higher CRP values, however this association has not been specifically studied in a large hypertensive cohort [22–24]. A local South African study demonstrated significantly higher CRP values in COVID-19 participants who demised, however failed to establish a direct link between CRP concentrations and mortality through multivariate analysis [25]. This is in keeping with another local study by Zemlin et al. which reported increased CRP levels among fatalities, albeit without significant prognostic value [26]. Once again, both these studies did not specifically analyse trends in hypertensive patients with COVID-19. In contrast, a study by Ozdemir et al. is one such study that specifically analysed the inflammatory response in hypertensive COVID-19 patients by investigating the prognostic value of the CRP/albumin ratio [27]. This ratio was found to be significantly higher in the non-survivor group and demonstrated promising mortality predictive ability after ROC curve analysis [27]. Another small study by Amar et al. comprising 129 participants showed that CRP level predicted a worse prognosis in hypertensive COVID-19 patients but not those without hypertension [28]. CRP is a well-documented marker of systemic inflammation and possibly represents the extent of the cytokine storm in patients with severe COVID-19 [29]. Inflammation is postulated to play a major role in mediating the adverse effects of SARS-CoV-2 infection. One such mechanism is thought to be thrombo-inflammation with dysregulation of the normal antithrombotic function of the endothelium in response to inflammatory stress, leading to inappropriate activation of platelets and the coagulation system with enhanced microvasculature thrombosis [29]. Thus, an elevated CRP not only indicates individuals at higher risk for severe disease and mortality, but potentially also identifies patients more likely to benefit from corticosteroid therapy. Other biomarkers of inflammation, such as IL-6, have also demonstrated prognostic significance in COVID-19, however CRP has the advantage of widespread availability and cost-effectiveness [29].

In our study, hypertensive patients with COVID-19 that received invasive mechanical ventilation were more than five times more likely to demise compared to those that did not (*p* = 0.004). This is not unsurprising and likely represents advanced disease. Interestingly, although a significantly greater number of patients with hypertension received oxygen and corticosteroid therapy compared to non-hypertensives, the number of participants requiring mechanical ventilation did not differ significantly between these two groups. Nonetheless, mechanical ventilation was an independent risk factor for mortality in the hypertensive cohort.

The link between hypertension-mediated organ damage (HMOD) and mortality due to COVID-19 remains underexplored. In our study, the presence of HMOD, the number of target organs affected, and the specific organ system affected differed significantly between survivors and non-survivors. Notably, those who did not survive were more frequently afflicted with HMOD and exhibited damage across a broader range of target organs. Specifically, ischemic heart disease and nephropathy were markedly more common among non-survivors, unlike peripheral vascular disease, cerebrovascular disease, and hypertensive retinopathy, which showed no significant difference in prevalence between survivors and non-survivors. Remarkably, the presence of ischemic heart disease increased the mortality risk more than eightfold (*p* = 0.033). In addition, patients with cerebrovascular disease (aOR 10.688; *p* = 0.052) and nephropathy (aOR 6.894 *p*= 0.057) were also found to have greater odds of mortality. This is in keeping with a large French study whereby stroke, ischemic heart disease and chronic kidney disease were all associated with a higher risk of hospitalisation and in-hospital mortality [30]. In addition the presence of peripheral vascular disease was also noted to be significantly associated with mortality which is contrary to our findings [30]. Uncontrolled hypertension is known to cause endothelial dysfunction through oxidative stress and inflammation [31]. Similarly, SARS-CoV-2 can trigger vascular inflammation, either by directly engaging ACE2 receptors or via an induced systemic inflammatory response, leading to endothelitis. It is hypothesized that the dual burden of hypertension and COVID-19 exacerbates endothelial damage, particularly in individuals with pre-existing vascular impairments, thus escalating the risk of severe disease [31].

This study underscores the significant impact of hypertension on COVID-19 outcomes within the South African context, highlighting that hypertensive patients exhibit a heightened risk of severe disease and mortality when infected with SARS-CoV-2 compared to non-hypertensives. Key findings include a higher prevalence of clinical and laboratory markers indicative of severe COVID-19, an increased necessity for oxygen and steroid therapy, and notably higher mortality rates among the hypertensive cohort. Independent risk factors for in-hospital mortality among these patients include lower oxygen saturation, higher pulse pressure, elevated C-reactive protein (CRP) levels, the requirement for mechanical ventilation, and the presence of ischemic heart disease, all suggesting that hypertension exacerbates COVID-19 severity. These results stress the critical need for early risk stratification and targeted management in hypertensive patients with COVID-19, particularly in settings like South Africa where hypertension prevalence is high, and healthcare resources may be limited. The study not only contributes to a deeper understanding of the interplay between hypertension and COVID-19 in an African population but also calls for enhanced pandemic preparedness and tailored treatment strategies to mitigate risks associated with this comorbidity.

### Study limitations

This study acknowledges several limitations that could impact its findings. Firstly, its retrospective design may introduce biases related to data collection and patient selection. The reliance on medical records for data extraction could lead to potential inaccuracies in the documentation of comorbidities, treatment interventions, and outcomes. Additionally, the study was conducted in a single tertiary hospital setting, which may limit the generalisability of the results to other healthcare settings or regions within South Africa. The study's sample size, although substantial, might not fully capture the diversity and complexity of hypertension's impact on COVID-19 outcomes across different demographic groups. Moreover, the exclusion of pregnant patients and those lacking necessary information could further constrain the study's comprehensiveness. Lastly, the dynamic nature of the COVID-19 pandemic, with evolving treatment protocols and the emergence of new virus variants, may affect the applicability of the study's conclusions over time.

## Conclusion

In conclusion, our study illustrates the exacerbated risk and severity of COVID-19 among hypertensive patients within the South African context, reinforcing hypertension as a critical comorbidity. The findings reveal significant associations between hypertension and increased COVID-19 morbidity and mortality, highlighting the urgent need for targeted interventions and management strategies for hypertensive individuals during the pandemic. This research not only fills a vital gap in understanding the dynamics between hypertension and COVID-19 outcomes in an African setting but also emphasises the necessity for heightened pandemic preparedness and healthcare resource allocation to mitigate the compounded risk presented by these concurrent health crises.

## Data Availability

The datasets used and/or analysed during the current study are available from the corresponding author upon reasonable request.

## Abbreviations

COVID-19: Coronavirus Disease 2019
SARS-CoV-2: Severe Acute Respiratory Syndrome Coronavirus 2
LMICs: Low- and Middle-Income Countries
ACE-2: Angiotensin-Converting Enzyme 2
ARBs: Angiotensin Receptor Blockers
ACEIs: Angiotensin-Converting Enzyme Inhibitors
HMOD: Hypertension-Mediated Organ Damage
PCR: Polymerase Chain Reaction
ICU: Intensive Care Unit
CRP: C-reactive Protein
SD: Standard Deviation
IQR: Interquartile Range
OR: Odds Ratio
CI: Confidence Interval
SPSS: Statistical Package for the Social Sciences
KEH: King Edward VIII Hospital
BREC: Biomedical Research Ethics Committee
COPD: Chronic Obstructive Pulmonary Disease
